# Combined assessment of the GAP index and body mass index at antifibrotic therapy initiation for prognosis of idiopathic pulmonary fibrosis

**DOI:** 10.1038/s41598-021-98161-y

**Published:** 2021-09-17

**Authors:** Yuzo Suzuki, Kazutaka Mori, Yuya Aono, Masato Kono, Hirotsugu Hasegawa, Koshi Yokomura, Hyogo Naoi, Hironao Hozumi, Masato Karayama, Kazuki Furuhashi, Noriyuki Enomoto, Tomoyuki Fujisawa, Yutaro Nakamura, Naoki Inui, Hidenori Nakamura, Takafumi Suda

**Affiliations:** 1 Second Division, Department of Internal Medicine, Hamamatsu University School of Medicine, 1-20-1 Handayama Higashi-ku, Hamamatsu, Shizuoka 431-3192 Japan; 2 Department of Respiratory Medicine, Shizuoka City Shimizu Hospital, Shizuoka, Japan; 3 Department of Respiratory Medicine, Seirei Hamamatsu General Hospital, Hamamatsu, Japan; 4 Department of Respiratory Medicine, Seirei Mikatahara General Hospital, Hamamatsu, Japan

**Keywords:** Respiratory tract diseases, Translational research

## Abstract

Antifibrotic therapy (AFT) slows disease progression in patients with idiopathic pulmonary fibrosis (IPF). The Gender-Age-Physiology (GAP) index, was developed based on data at IPF diagnosis before the introduction of AFT and has not been evaluated in the AFT context. Further, recent advances have revealed the importance of body-composition factors in prognosis of IPF treated with AFT. This multi-centre, retrospective study aimed to evaluate the GAP index and body mass index (BMI) at the time of AFT initiation for predicting prognosis in patients with IPF. This study included two patient cohorts of IPF receiving AFT, Hamamatsu cohort (n = 110) and Seirei cohort (n = 119). The distribution of GAP stages I, II, and III was 38.2%, 43.6%, and 18.2%, respectively, in Hamamatsu cohort; in Seirei cohort, it was 41.2%, 50.4%, and 8.4%, respectively. In both cohorts, the GAP index distinctly classified prognosis into three groups (log-rank test). Interestingly, a lower BMI showed prognostic value independent of the GAP index in multivariate analyses. Subsequently, combining the GAP index with BMI at AFT initiation successfully divided the patients with IPF into four distinct prognoses. Assessment of the GAP index and BMI measurement at AFT initiation are important for predicting prognosis in patients with IPF.

## Introduction

Idiopathic pulmonary fibrosis (IPF) is a fibrotic progressive interstitial lung disease (ILD) characterised by declined pulmonary function and overall poor prognosis^1,2^. The Gender-Age-Physiology (GAP) index, a simple point-scoring calculator of multidimensional prognostic staging system, was originally proposed and validated in 2012 for the prediction of 1, 2 and 3-year mortalities in IPF^3^. The GAP index calculates the baseline IPF characteristics and has shown excellent prognostic-group separation ability.

After development of the GAP index, antifibrotic therapy (AFT), i.e. pirfenidone and nintedanib, was established and recommended for IPF treatment in the international guideline of 2015^2^. The pirfenidone and nintedanib slow disease progression by reducing the annual decline of forced vital capacity (FVC) in patients with IPF^4–7^. Further, AFT was reported to reduce the decline of FVC in patients with other ILD types, including systemic sclerosis-associated ILD^8^ and progressive fibrosing ILDs^9,10^. Based on its documented ability to delay lung-function deterioration, AFT is considered to reduce the risk of mortality^11,12^. However, the utility of the GAP index in the context of AFT has not been fully evaluated, and the development of a simple and easily applicable prognostic staging system for AFT is expected.

A lower body mass index (BMI) was previously reported to be associated with poor outcome in patients with IPF. Recently, the clinical implication of sarcopenia, which is characterised by progressive and generalised skeletal disorder involving accelerated loss of muscle mass and function, has been highlighted in various diseases^14,15^. These metabolic dysfunctions, partly represented as muscle wasting and body-weight loss, are frequently found in patients with various respiratory-disease types, including ILD^16–19^. Importantly, muscle wasting and body-weight loss have been associated with poor outcome in patients with IPF^13,18–21^. These previous studies suggested that preventing skeletal-muscle wasting as well as preserving body weight and lung function are important for the management of patients with IPF.

This multi-centre, retrospective, two-cohort study aimed to evaluate the GAP index at AFT initiation in patients with IPF. Additionally, this study also assessed the value of the BMI and of combined BMI and GAP index assessment for prognosis discrimination in patients with IPF treated with AFT.

## Results

### Clinical characteristics

**Figure 1:**
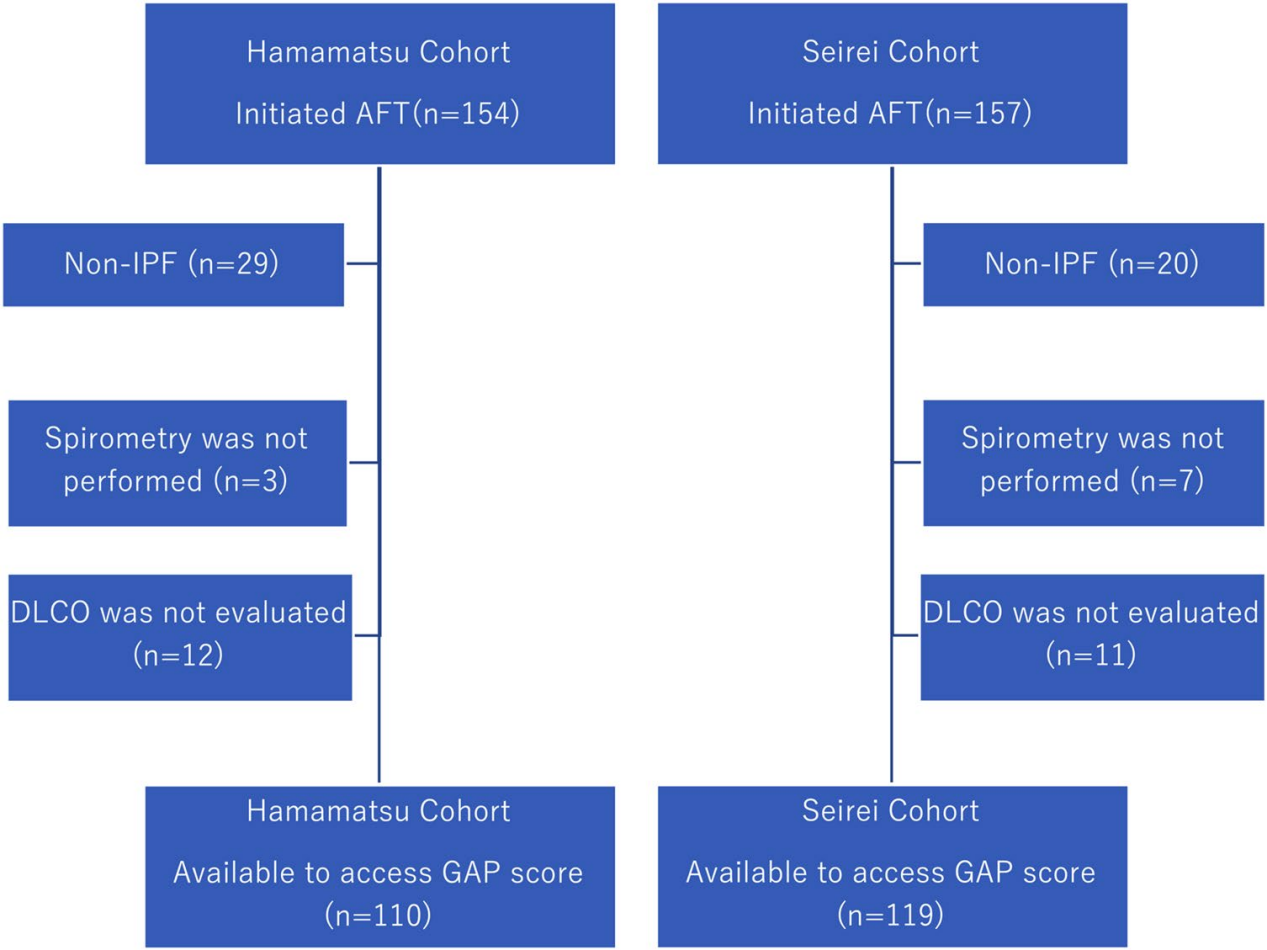
Flow diagram of patient selection. *AFT* antifibrotic therapy, *IPF* idiopathic pulmonary fibrosis, *DLCO* diffuse capacity of the lung for carbon monoxide.

The clinical characteristics of the patients with IPF at the time of AFT initiation are shown in Table 1 and Fig. 1. Most patients were approximately 70 years old and 80% of the patients were male in both cohorts. Pirfenidone was commonly used in the Hamamatsu cohort, and surgical lung biopsy was frequently performed in the Hamamatsu cohort. The median follow-up period since AFT was initiated was approximately 2 years, with 70 patients having more than a 3-year observation period and 43 patients having more than a 5-year observation period. The number of patients with previous history of AE at AFT initiation tended to be higher in the Seirei cohort: 16 (13.4%) vs. 7 (6.4%), respectively. The pulmonary function test showed severe-to-moderate impairment of spirometry and decreased %DLCO in both cohorts. The levels of serum albumin were slightly lower in the patients in the Seirei cohort. Long-term oxygen therapy (LTOT) and immunosuppressants were more frequently prescribed in the Seirei cohort. Immunosuppressants were mainly initiated due to acute exacerbation (AE), and some were prescribed before the PANTHOR trial^22^.

**Table 1:** Clinical characteristics of 229 patients with IPF at initiations of antifibrotic therapy.

	IPF combined cohort (n = 229)	Hamamatsu cohort (n = 110)	Seirei cohort (n = 119)	p-value (Hamamatsu cohort vs Seirei cohort)
Age, year	72.0 [67.5–72.0]	72.0 [68.0–75.3]	73.0 [67.0–76.0]	0.8259
Sex, male/female	186 (81.2%)/43 (18.8%)	91 (82.7%)/19 (17.3%)	95 (79.8%)/24 (20.2%)	0.6144
Surgical lung biopsy	50 (21.8%)	35 (31.8%)	15 (12.6%)	0.0007
Diagnosis ~ antifibrotic	13.3 [2.5–45.9]	17.9 [2.2–57.2]	11.5 [2.8–39.1]	0.2081
Follow-up period (anti fibrotic ~)	24.3 [10.8–38.0]	25.8 [12.4–37.9]	21.1 [9.6–38.8]	0.2473
Pirfenidone/nintedanib	140 (61.1%), 89 (38.9%)	76 (69.1%), 34 (30.9%)	64 (53.8%), 55 (46.2%)	0.0211
History of AE	23 (10.0%)	7 (6.4%)	16 (13.4%)	0.0825
Never/former & current smoker	50 (21.8%), 179 (78.2%)	25 (22.7%), 85 (77.3%)	25 (21.0%), 94 (79.0%)	0.8729
Smoking pack-year	30.0 [2.6–48.8]	40.0 [18.0–59.0]	40.0 [18.0–59.0]	0.8122
BMI, kg/m^2^	23.0 [21.1–25.4]	23.3 [21.4–25.7]	22.8 [20.7–25.3]	0.2837
**Pulmonary function test**				
FVC, %-pred	68.3 [57.0–80.7]	67.2 [55.7–80.4]	69.1 [58.1–81.8]	0.4463
FEV_1_, %-pred	74.2 [64.1–90.4]	70.7 [59.1–82.9]	79.4 [69.0–93.5]	< 0.0001
FEV_1_/FVC, %	85.4 [79.8–91.7]	85.0 [78.5–90.9]	88.0 [80.4–92.9]	0.0759
DLCO, %	59.0 [44.4–71.3] (n = 217)	54.8 [42.4–71.7] (n = 102)	60.8 [45.5–71.2] (n = 115)	0.1676
**Laboratory**				
Hb, g/dl	13.6 [12.5–14.7]	13.5 [12.2–14.6]	13.6 [12.7–14.8]	0.2876
TP, g/dl	7.4 [7.0–7.8]	7.5 [7.1–7.8]	7.4 [6.9–7.9]	0.9728
Alb, g/dl	3.9 [3.6–4.1]	4.0 [3.7–4.2]	3.8 [3.5–4.0]	0.0010
LDH, U/L	230 [203–272]	237 [204–273]	225 [203–270]	0.4627
CRP, mg/dl	0.2 [0.1–0.5]	0.2 [0.1–0.5]	0.2 [0.1–0.5]	0.6172
KL-6, U/ml	1102 [795–1475]	1018 [768–1432]	1169 [855–1673]	0.0507
SP-D ng/ml	247 [157–362]	240 [152–347]	256 [158–395]	0.2178
**Treatment**				
No treatment	145 (63.3%)	81 (73.6%)	64 (53.8%)	0.0025
LTOT	73 (31.9%)	28 (25.5%)	45 (37.8%)	0.0482
Immunosuppressants	39 (17.0%)	11 (10.0%)	28 (23.5%)	0.0080

*AE* acute exacerbation, *BMI* body mass index, *FVC* forced vital capacity, *FEV*_*1.0*_ forced expiratory volume in 1.0 s, *DLCO* diffuse capacity of the lung for carbon monoxide, *KL-6* Krebs von den Lunge-6, *SP-D* surfactant protein-D, *LTOT* long-term oxygen therapy.

### Assessment of the GAP stage in patients with IPF at the time of AFT initiation

The distributions of patients with IPF at the time of AFT initiation according to the GAP index are shown in Table 2. The frequencies of GAP stages I and stage II were approximately 40%. Among them, GAP stage II was most frequent in both cohorts. Meanwhile, the proportion of GAP stage III was lower than 20% in both cohorts, and the proportion of GAP stage III was lower in the Seirei cohort than in the Hamamatsu cohort; especially, that in the Seirei cohort was 8.4%.

**Table 2:** GAP stage in patients with IPF at initiations of antifibrotic therapy.

	IPF combined cohort (n = 229)	Hamamatsu cohort (n = 110)	Seirei cohort (n = 119)	p-value (Hamamatsu cohort vs Seirei cohort)
Stage I	91 (39.7%)	42 (38.2%)	49 (41.2%)	0.6862
Stage II	108 (47.2%)	48 (43.6%)	60 (50.4%)	0.3540
Stage III	30 (13.1%)	20 (18.2%)	10 (8.4%)	0.0321

*GAP* Gender-Age-Physiology.

### Prognostic classification of the GAP index in patients with IPF treated with AFT

**Figure 2:**
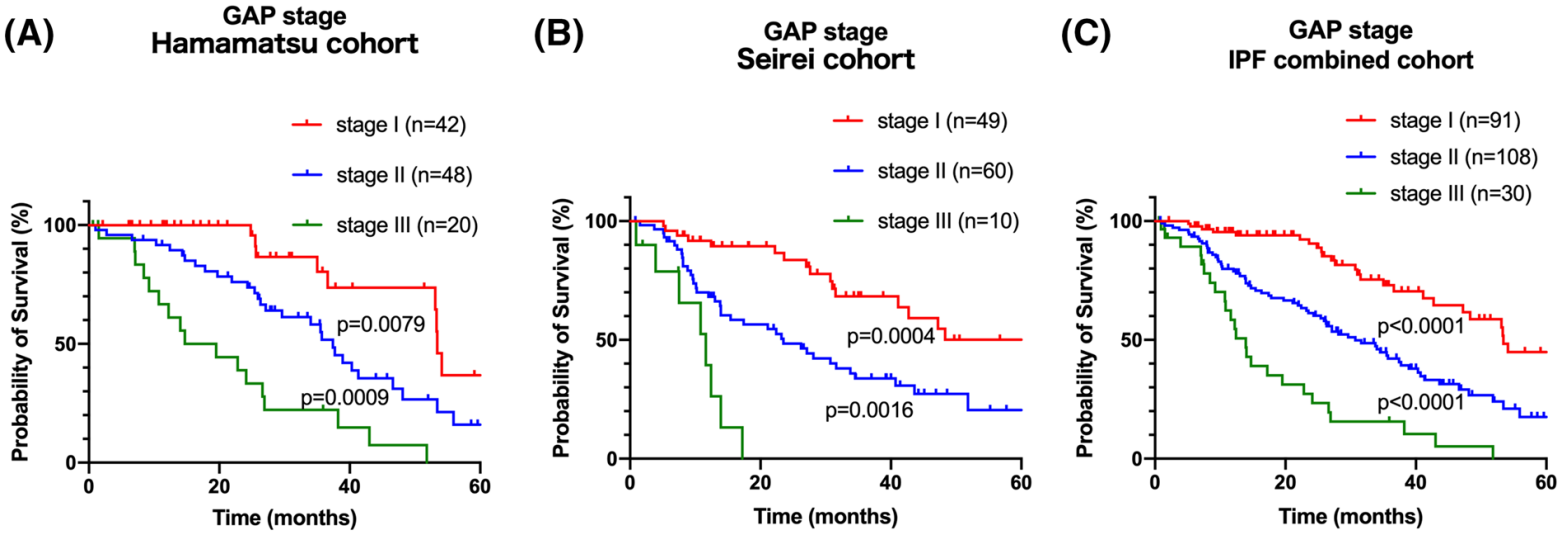
Prognostic classifications according to the GAP index at initiation of antifibrotic therapy in patients with IPF. Kaplan–Meier curves based on the data of the patients with IPF of the Hamamatsu cohort **(A)**, Seirei cohort **(B)**, and combined cohort **(C)** according to the GAP stage. P values were determined using the log-rank test. *GAP* Gender-Age-Physiology, *IPF* idiopathic pulmonary fibrosis.

**Figure 3:**
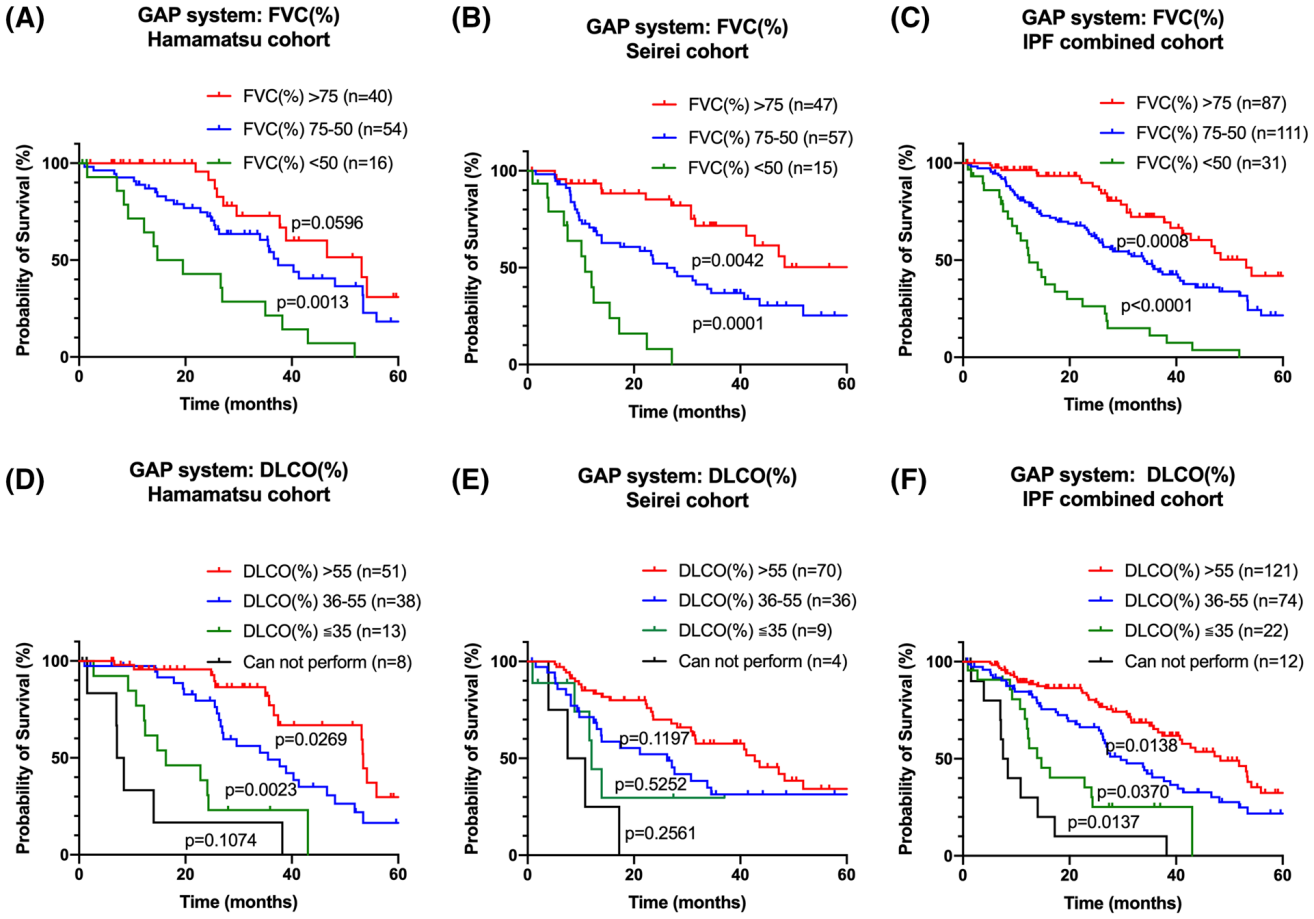
Prognostic classifications according to GAP index: FVC (%) and GAP index: DLCO (%). Kaplan–Meier curves based on the data of the patients with IPF of the Hamamatsu cohort **(A,D)**, Seirei cohort **(B,E)**, and combined cohort **(C,F)** according to GAP stage: FVC (%) and GAP index: DLCO (%). P values were determined using the log-rank test. *GAP* Gender-Age-Physiology, *FVC* forced vital capacity, *DLCO* diffuse capacity of the lung for carbon monoxide, *IPF* idiopathic pulmonary fibrosis.

During the follow-up period, 122 deaths were noted. There were no significant differences in causes of death between the two cohorts (Table 3). In both cohorts, approximately 60% of patients died because of chronic respiratory failure, whereas the incidence of lung cancer was lower than 10%. The survival analyses according to the GAP index are shown in Fig. 2 and Supplementary-Table S1. In both cohorts, the GAP index successfully divided the prognosis of patients with IPF treated with AFT into three groups with distinct prognosis. The discrimination performance of the GAP index in patients of the combined cohorts was 0.675 (C-statistics). Among the components of the GAP index, FVC (%) and DLCO (%) also yielded prognostic separation in the combined-cohort patients (Fig. 3), but not the ‘Age’ and ‘Gender’ factors (not shown). The C-index of the ‘GAP index: FVC (%)’ and ‘GAP index: DLCO (%)’ were 0.668 and 0.655, respectively.

**Table 3:** Cause of mortality in patients with IPF treated with antifibrotic therapy.

	IPF combined cohort (n = 229)	Hamamatsu cohort (n = 110)	Seirei cohort (n = 119)	p-value (Hamamatsu cohort vs Seirei cohort)
Chronic respiratory failure	70 (57.4%)	33 (57.9%)	37 (56.9%)	1.0000
Acute exacerbation	29 (23.8%)	15 (26.3%)	14 (21.5%)	0.6704
Lung cancer	7 (5.7%)	2 (3.5%)	5 (7.7%)	0.4467
Pneumothorax	5 (4.1%)	4 (7.0%)	1 (1.5%)	0.1839
Infection	3 (2.5%)	1 (1.8%)	2 (3.1%)	1.0000
Others	8 (6.6%)	2 (3.5%)	6 (9.2%)	0.2813

### Prognostic factors in patients with IPF treated with AFT

The univariate and multivariate analyses are presented in Table 4. As shown, the GAP index and its components, %FVC and %DLCO, were found to be significant prognostic factors in the multivariate analyses. Additionally, a lower BMI was a significant prognostic factor independent of the GAP index in the multivariate analyses.

**Table 4:** Prognostic factors in 229 patients with IPF treated with antifibrotic therapy by univariate and multivariate Cox-proportion analyses.

Predictor	HR	95% CI	p-value		HR	95% CI	p-value
**Univariate analysis**				**Multivariate analysis 1**			
Age, year	1.022	0.995–1.051	0.1169	Age, year	0.998	0.970–1.028	0.8877
Gender, male	1.262	0.767–1.981	0.3324	Gender, male	1.243	0.734–2.241	0.4428
History of AE, yes	2.277	1.314–3.704	0.0017	History of AE, yes	1.320	0.710–2.313	0.3541
Pirfenidone	1.039	0.703–1.566	0.8519	BMI, kg/m^2^ (continuous variable)	0.929	0.869–1.077	0.0273
BMI, kg/m^2^ (continuous variable)	0.896	0.847–0.947	0.0001	FVC, %	0.984	0.971–0.997	0.0199
FVC, %	0.967	0.956–0.978	< 0.0001	DLCO, %	0.983	0.970–0.996	0.0119
FEV_1_, %	0.986	0.975–0.996	0.0093	LTOT, yes	1.692	1.104–2.562	0.0141
FEV_1_/FVC, %	1.065	1.040–1.091	< 0.0001	**Multivariate analysis 2**			
DLCO, %	0.974	0.962–0.986	< 0.0001	GAP index, stage	2.161	1.615–2.899	< 0.0001
TP, g/dl	0.960	0.733–1.277	0.7720	BMI, kg/m^2^ (continuous variable)	0.931	0.882–0.983	0.0093
Alb, g/dl	0.572	0.376–0.881	0.0094	History of AE, yes	1.354	0.758–2.289	0.2799
KL-6, U/ml	1.000	1.000–1.001	< 0.0001	LTOT, yes	1.706	1.138–2.533	0.0088
SP-D, ng/ml	1.001	1.000–1.002	0.0760	**Multivariate analysis 3**			
LTOT, yes	2.660	1.847–3.810	< 0.0001	GAP index: gender	1.303	0.801–2.214	0.3046
GAP index, stage	2.637	1.993–3.491	< 0.0001	GAP index: age	1.021	0.757–1.426	0.8990
GAP index, score	1.641	1.437–1.870	< 0.0001	GAP index: FVC%	1.666	1.201–2.321	0.0024
GAP index: age	1.226	0.917–1.701	0.1945	GAP index: DLCO%	1.495	1.170–1.907	0.0012
GAP index: FVC%	2.549	1.913–3.402	< 0.0001	BMI, kg/m^2^ (continuous variable)	0.930	0.879–0.983	0.0104
GAP index: DLCO%	1.969	1.591–2.419	< 0.0001	History of AE, yes	1.414	0.785–2.414	0.2242
BMI < 24.0 kg/m^2^ (categorical variable)	2.110	1.440–3.157	0.0002	LTOT, yes	1.519	0.983–2.317	0.0556

*AE* acute exacerbation, *BMI* body mass index, *FVC* forced vital capacity, *FEV*_*1.0*_ forced expiratory volume in 1.0 s, *DLCO* diffuse capacity of the lung for carbon monoxide, *KL-6* Krebs von den Lunge-6, *SP-D* surfactant protein-D, *LTOT* long-term oxygen therapy, *GAP* Gender-Age-Physiology.

### Prognostic value of the BMI at AFT initiation in patients with IPF

**Figure 4:**
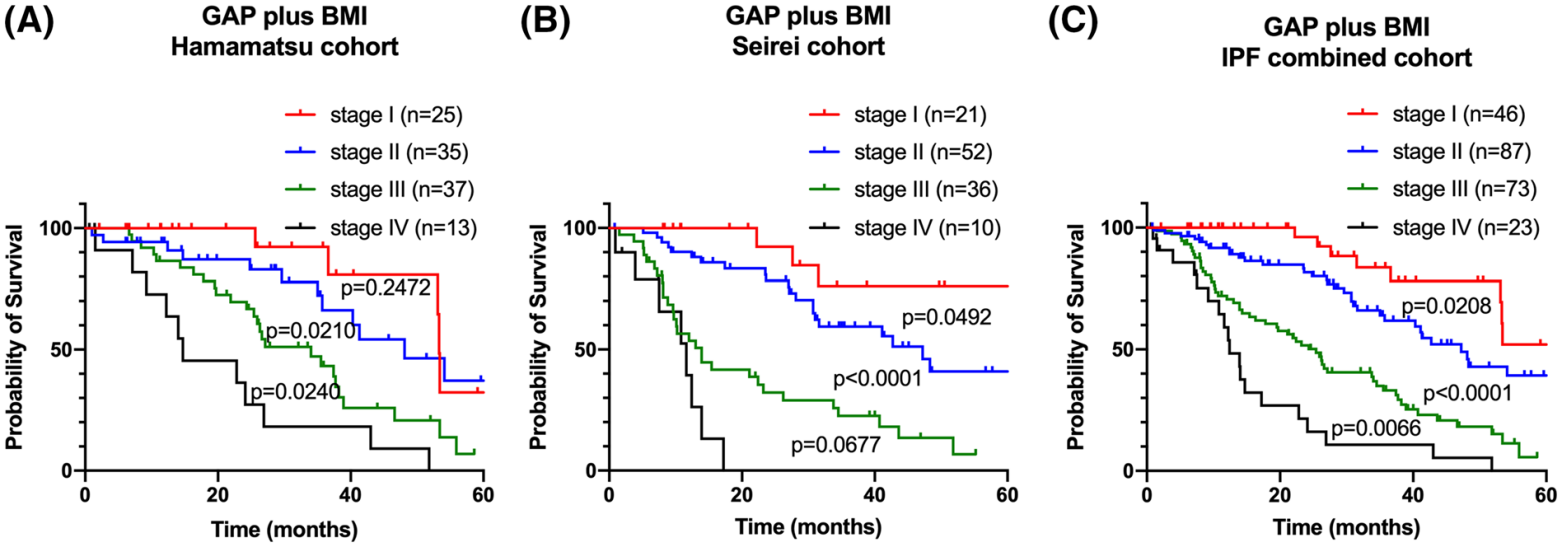
Prognostic classifications according to GAP plus BMI. Kaplan–Meier curves based on the data of the patients with IPF of the Hamamatsu cohort **(A)**, Seirei cohort **(B)**, and combined cohort **(C)** according to GAP plus BMI. P values were determined using the log-rank test. *GAP* Gender-Age-Physiology, *BMI* body mass index, *IPF* idiopathic pulmonary fibrosis.

As the BMI showed prognostic value independent of the GAP index, we performed combined assessment of the GAP index with the BMI, i.e., GAP plus BMI, for IPF prognosis. Interestingly, a reverse J-shaped association was seen between the BMI and mortality rate in the Cox proportional hazards regression model (Supplementary-Figure S1). According to the ROC analyses, we identified a BMI 24 kg/m^2^ as the optimal cut-off. If the value of the BMI was lower than the cut-off value, the severity of the GAP stage was advanced by one stage. For example, the patients with GAP stage I who had BMI < 24 kg/m^2^ were categorized as GAP plus BMI stage II. Subsequently GAP plus BMI classified patients into four groups. The survival analyses according to GAP plus BMI are shown in Fig. 4. For clinical relevance, survival curves applying BMI < 20 kg/m^2^ (C-statistics 0.688) were also shown in Supplementary-Figure S2. GAP plus BMI was successful in distinguishing the prognoses in the combined-cohort patients with IPF (C-statistics 0.698). To calculate model improvement by GAP plus BMI at the 1-year, 3-year, and 5-year survivals, we applied IDI and NRI analyses. GAP plus BMI improved the discriminative performance of 3-year survival at 21% compared to that provided by the GAP index alone, although statistical significance was not reached (Table 5).

**Table 5:** Additional predictive values of GAP plus BMI at initiations of antifibrotic therapy in patients with IPF.

	C-index [95% CI]		NRI [95% CI]		IDI [95% CI]	
			Estimate	p-value*	Estimate	p-value*
GAP index: stage	0.675 [0.630–0.720]		–	–	–	–
GAP index: FVC (%)	0.668 [0.624–0.713]	1-year survival	−0.256 [−0.366 to 0.304]	0.711	−0.006 [−0.056 to 0.041]	0.844
		3-year survival	−0.197 [−0.311 to 0.288]	0.977	−0.005 [−0.066 to 0.066]	0.983
		5-year survival	−0.152 [−0.706 to 0.372]	0.485	−0.097 [−0.224 to 0.007]	0.080
GAP index: DLCO (%)	0.655 [0.603–0.707]	1-year survival	−0.125 [−0.331 to 0.096]	0.179	0.010 [−0.029 to 0.066]	0.691
		3-year survival	−0.087 [−0.290 to 0.070]	0.279	−0.020 [− 0.089 to 0.047]	0.578
		5-year survival	−0.477 [−0.652 to −0.202]	0.007	−0.158 [−0.244 to −0.066]	0.007
GAP plus BMI	0.698 [0.652–0.743]	1-year survival	0.209 [−0.015 to 0.355]	0.073	0.012 [−0.023 to 0.045]	0.465
		3-year survival	0.210 [−0.001 to 0.361]	0.053	0.032 [−0.016 to 0.078]	0.206
		5-year survival	0.234 [−0.110 to 0.492]	0.193	0.016 [−0.075 to 0.099]	0.718

*Compared with GAP index.

*CI* confidence interval, *NRI* net reclassification improvement, *IDI* integrated discrimination improvement, *GAP* Gender-Age-Physiology, *FVC* forced vital capacity, *DLCO* diffuse capacity of the lung for carbon monoxide, *BMI*; body mass index.

## Discussion

The present study retrospectively examined the utility of the GAP index and of the BMI for prognosis prediction in patients with IPF treated with AFT. First, the GAP index at AFT initiation yielded clear prognostic distinction in the two cohorts of patients with IPF. Next, as BMI showed prognostic value independent of the GAP index, we evaluated the prognosis using the GAP index in conjunction with BMI: GAP plus BMI. Assessment of GAP plus BMI at AFT initiation successfully divided the patients into four groups with distinct prognoses. Our data suggested the clinical usefulness of the GAP index and importance of BMI assessment at AFT initiation in patients with IPF.

The GAP index was developed as a clinical baseline prediction model to distinguish prognostic groups in IPF based on data obtained at the time of diagnosis^3^. The GAP index is an easy and simple method that enables the assessment of cross-sectional data, without need for longitudinal data, which is advantageous. Accordingly, the GAP index is widely used and its utility has been shown in other types of ILD including unclassifiable ILD, chronic hypersensitive pneumonitis, and connective tissue disease-associated ILD^23,24^. However, at the time of GAP index development, no pharmacological therapy including pirfenidone was recommended in the international guideline^25^. Thus, the clinical implications of the GAP index in AFT have not been fully evaluated. In this regard, this study examined the prognostic classifications of the GAP index with two cohorts of patients with IPF treated with AFT and showed that assessment of the GAP index at AFT initiation clearly distinguished the prognoses.

The most common cause of mortality in these cohorts was chronic respiratory failure (57.4%) followed by AE (23.8%), whereas the lung-cancer incidence was very low at 5.7%. Interestingly, an epidemiological study conducted in Japan between 2003 and 2007 (i.e. before the introduction of AFT) reported the incidence of AE, chronic respiratory failure, and lung cancer at 40%, 24%, and 11%, respectively^26^; the lung-cancer incidence was twice as high as that in our cohort and resulted in an increase in the combined incidence of chronic respiratory failure and AE after AFT development. Although AE can occur in patients with preserved lung function, low FVC was reported as the most consistent risk factor for AE in IPF, and low DLCO was also identified as a well-known risk factor for AE in an international statement^27^. Therefore, these changes in cause of death may have positively contributed to mortality prediction using the GAP index at AFT initiation.

Given that the GAP index is a baseline risk-prediction model largely based on physiological factors, these issues also imply their own limitations. Disease behaviour or exercise capacity are not required to calculate the GAP stage. Indeed, both baseline and changes in spirometry and 6-min walking test (6MWT) were reported to be independent predictors of mortality^28,29^. Additionally, an overestimated risk for mortality with the GAP index was reported by the same authors who developed the GAP index^30^. They reported improvement in risk prediction performance using a modified GAP index that also included longitudinal change in FVC and respiratory hospitalization^30^. However, assessing longitudinal variables and 6MWT may not be suitable for risk assessment in severe patients or patients for whom there are no historical data available.

In this setting, as the BMI was shown to have prognostic value independent of the GAP index and it is easy to measure, the present study examined the value of using GAP plus BMI in patients with IPF treated with AFT for prognostic separations. Recently, clinical implications of sarcopenia, a metabolic dysfunction involving loss of skeletal muscle, in ILD was reported beyond those in chronic obstructive pulmonary disease and cancer^17–19^. Indeed, skeletal-muscle loss was well correlated with lower BMI and was associated with worse outcome in patients with IPF^18,19^. Thus, lower BMI partly represents skeletal-muscle wasting in patients with IPF. Further, consistent with our findings, a second analysis of the INPULSIS study reported that lower BMI (< 25 kg/m^2^) and weight loss (> 5% during 52 weeks) were associated with faster decline in FVC, suggesting shorter survivals in such patients^20^. Although GAP plus BMI did not achieve significant improvement of model discrimination compared with that of the original GAP index, GAP plus BMI distinguished patients into four distinct prognoses. Especially, patients with GAP stages I and II (approximately 85% of our cohort) were re-classified from two groups into three groups. This multidimension approach might be helpful for physicians in practice.

The present study had several limitations. Although we confirmed the utility of the GAP index at AFT initiation using two cohorts of patients with IPF, this study was retrospective. Further, there had been reported concerns regarding the use of p-value in net reclassification improvement and its validity^31^. Second, the sample size was not sufficiently large. Given the small number of patients in each cohort, significance of GAP plus BMI was ascertained when we examined the two cohorts together. Third, the cut-off of the BMI appears to depend on ethnicity and country^20^. In our cohort, although few patients were obese, reverse-J associations were found between BMI and mortality, suggesting that both upper and lower cut-offs in BMI might be needed. Indeed, sarcopenic obesity is known to lead to poor outcomes in patients with cancer^32^. Collectively, these limitations may have introduced potential biases to this study. Thus, large-scale prospective studies are required to overcome these limitations.

In conclusion, the present retrospective study showed that assessment of the GAP index at AFT initiation could successfully separate patients with IPF in prognostic groups. Our multivariate prognostic evaluation also revealed that lower BMI was associated with poor outcome independent of the GAP index. Interestingly, GAP plus BMI also separated patients into four distinct prognoses in the combined-cohort of patients. Collectively, these results indicated the value of the GAP index and BMI measurement for assessing prognostic prediction in patients with IPF treated with AFT.

## Methods

### Subjects

This retrospective study initially included 311 consecutive patients with ILD who started treatment with pirfenidone or nintedanib at Hamamatsu University of School of Medicine (Hamamatsu cohort, n = 154), Seirei Hamamatsu Hospital, and Seirei Mikatahara Hospital (Seirei cohort, n = 157). All patients were treated between February 2009 and March 2020. Eighty-two patients with ILD were excluded from the study: 49 patients were diagnosed with non-IPF ILD, ten patients with IPF did not undergo spirometry, and 23 were not evaluated with the diffusion capacity of the lung for carbon monoxide test (DLCO) at the time of AFT initiation. Thus, this study finally included 229 patients with IPF treated with AFT who had available data to assess the GAP score: the Hamamatsu cohort (n = 110) and Seirei cohort (n = 119) (Fig. 1). All participants fulfilled the IPF consensus criteria^1,25^. The study protocol was approved by the Ethical Committee of Hamamatsu University School of Medicine (17-196) and was carried out in accordance with the approved guidelines. The need for patient approval and/or informed consent was waived by the Ethical Committee of Hamamatsu University School of Medicine, because of the retrospective nature of the study.

### Data collection

Clinical data were obtained from the patients’ medical records. Laboratory findings and pulmonary and functional test results obtained at the time of AFT initiation were recorded. AE was diagnosed based on the ATS guidelines^27,33^.

### Assessing the GAP index and GAP plus BMI index

The GAP score was calculated based on data at the time of AFT initiation according to a previous study^3^: sex (female, 0 points; male, 1 point), age (≤ 60 years , 0 points; 61–65 years, 1 point; > 65 years, 2 points), %FVC (> 75%, 0 points; 50–75%, 1 point; < 50%, 2 points), and %DLCO (> 55%, 0 points; 36–55%, 1 point; ≤ 35%, 2 points; cannot perform, 3 points). The GAP stage was defined based on the total GAP score: stage I (0–3 points), stage II (4–5 points), and stage III (6–8 points). The GAP plus BMI was calculated based on the GAP stage and BMI at the time of AFT initiation. If the BMI value was lower than the cut-off value, the severity of the GAP stage was advanced by one stage.

### Statistical analysis

Discrete variables are expressed as totals (percentages), and continuous variables are expressed as the median [interquartile range]. The Mann–Whitney U test was used to compare continuous variables. Fisher’s exact test for independence was used to compare categorical variables. The area under the receiver operating characteristic (ROC) curve was used to identify the optimal cut-off for the BMI. The overall survival time was measured from AFT initiation. Univariate and multivariate analyses were also performed using a Cox proportional hazards regression model. Cumulative survival probabilities were calculated using the Kaplan–Meier method and the log-rank test. The model performance was evaluated by discrimination using concordance statistics (C-statistics), i.e., the ability of a model to discriminate those with an outcome from those without an outcome. Model improvement was calculated by comparison with the GAP index; change in C-statistics, integrated discrimination improvement (IDI), and net reclassification improvement (NRI) were employed. Statistical analyses were performed using JMP (Ver13, SAS Institute, Inc., Cary, NC) and R (Ver4.0.2, R Foundation for Statistical Computing, Vienna, Austria). All analyses were two-tailed, and P-values < 0.05 were considered statistically significant.

## Supplementary Information

Supplementary Information.

## Data Availability

The data that support the findings of this study are available from the corresponding authors upon reasonable request.

## Abbreviations

AFT: Antifibrotic therapy
BMI: Body mass index
DLCO: Diffusion capacity of the lung for carbon monoxide
FVC: Forced vital capacity
GAP: Gender-Age-Physiology
IDI: Integrated discrimination improvement
ILD: Interstitial lung disease
IPF: Idiopathic pulmonary fibrosis
NRI: Net reclassification improvement
ROC: Receiver operating characteristic
