# Supplementary material for: Combined assessment of the GAP index and body mass index at antifibrotic therapy initiation for prognosis of idiopathic pulmonary fibrosis
Source: Sci Rep. 2021 Sep 17;11:18579. doi: 10.1038/s41598-021-98161-y (PMC8448817; doi:10.1038/s41598-021-98161-y)
Supplement: Supplementary file 1 — Supplementary Information. [file 41598_2021_98161_MOESM1_ESM.pdf]

SUPPLEMENTARY INFORMATION

Combined Assessment of the GAP Index and Body Mass Index at Antifibrotic Therapy Initiation for Prognosis of Idiopathic Pulmonary Fibrosis

Authors' full names: Yuzo Suzuki, M.D., Ph.D.[1], Kazutaka Mori, M.D., Ph.D.[2], Yuya Aono, M.D.[1], Masato Kono, M.D., Ph.D.[3], Hirotsugu Hasegawa, M.D., Ph.D.[4], Koshi Yokomura, M.D., Ph.D.[4], Hyogo Naoi, M.D.[1], Hironao Hozumi, M.D., Ph.D.[1], Masato Karayama, M.D., Ph.D.[1], Kazuki Furuhashi, M.D., Ph.D.[1], Noriyuki Enomoto, M.D., Ph.D.[1], Tomoyuki Fujisawa, M.D., Ph.D.[1], Yutaro Nakamura, M.D., Ph.D.[1], Naoki Inui, M.D., Ph.D.[1], Hidenori Nakamura, M.D., Ph.D. [3], Takafumi Suda, M.D., Ph.D.[1]

Authors' affiliations:
[1]Second Division, Department of Internal Medicine, Hamamatsu University School of Medicine, Hamamatsu, Japan
[2]Department of Respiratory Medicine, Shizuoka City Shimizu Hospital, Shizuoka, Japan
[3]Department of Respiratory Medicine, Seirei Hamamatsu General Hospital, Hamamatsu, Japan
[4]Department of Respiratory Medicine, Seirei Mikatahara General Hospital, Hamamatsu, Japan

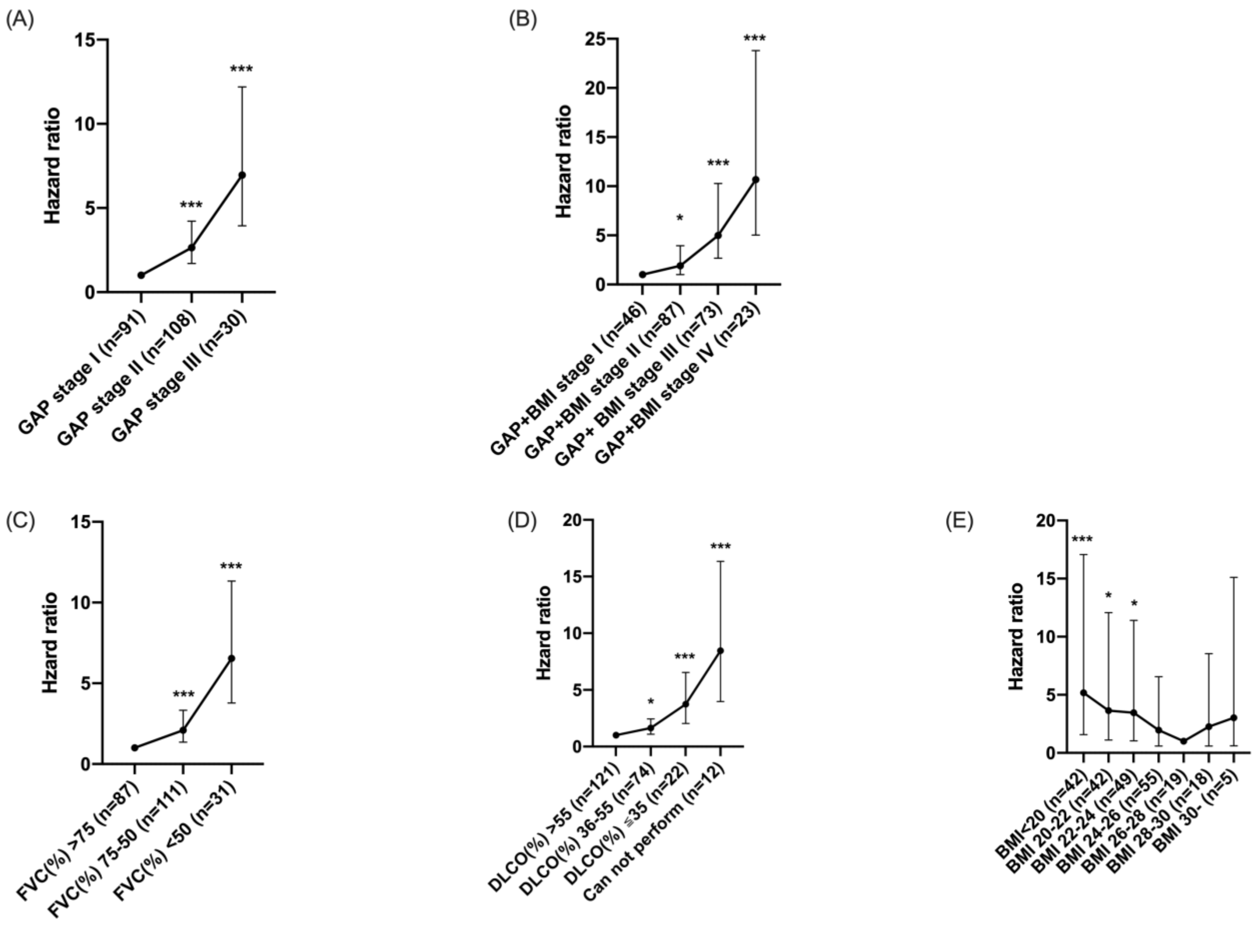

Supplementary-Figure S1. Hazard risk for mortality according to the GAP system and GAP plus BMI

Hazard risk for mortality according to the GAP system: stage (A), GAP plus BMI (B), GAP system: FVC (%) (C), GAP system: DLCO (%) (D), and BMI (E). P values were determined using a Cox proportional hazards regression model. *: P<0.05, ***: P<0.0001

GAP: Gender-Age-Physiology, BMI: body mass index, FVC: forced vital capacity, DLCO: diffuse capacity of the lung for carbon monoxide

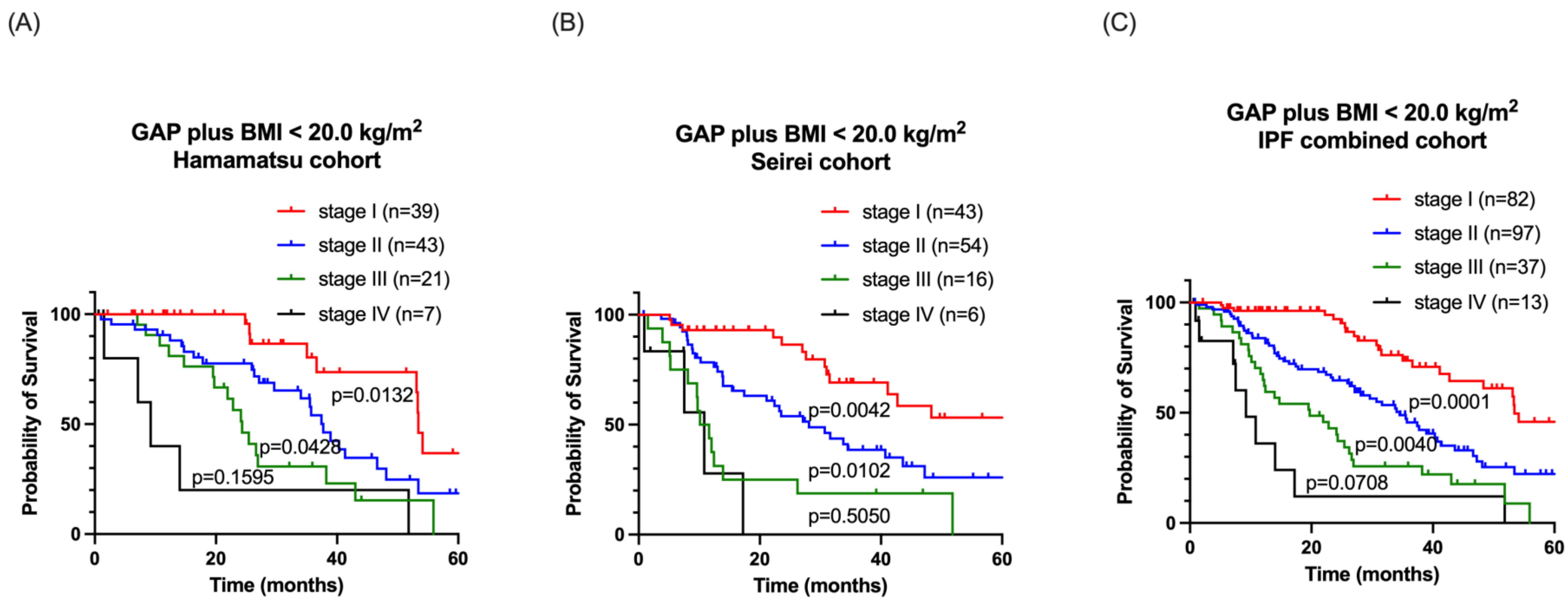

Supplementary-Figure S2. Prognostic classifications according to GAP plus BMI <20.0kg/m^2.

Kaplan–Meier curves based on the data of the patients with IPF of the Hamamatsu cohort (A), Seirei cohort (B), and combined cohort (C) according to GAP plus BMI <20.0kg/m^2. P values were determined using the log-rank test. GAP; Gender-Age-Physiology, BMI; body mass index, IPF: idiopathic pulmonary fibrosis

Supplementary-Table S1. Median and 3-years survivals of IPF patients treated with antifibrotic therapy

		IPF cohort (n=229)		Hamamatsu cohort (n=110)		Seirei cohort (n=119)	
GAP index	GAP stage I	53.4	(95.4%, 73.0%, 44.9%) *	53.4	(100%, 80.4%, 36.8%)	67.2	(91.7%, 68.3%, 50.1%)
	GAP stage II	30.7	(78.9%, 42.1%, 15.6%)	37.4	(91.6%, 51.7%, 16.6%)	23.5	(68.2%, 33.8%, 20.5%)
	GAP stage III	13.9	(58.4%, 15.6%, 0%)	17.1	(66.7%, 22.2%, 0%)	11.6	(39.4%, 0%, 0%)
GAP index: FVC (%)	FVC (%) > 75	53.1	(96.4%, 72.3%, 41.9%)	53.1	(100%, 72.8%, 30.9%)	67.2	(93.5%, 71.7%, 50.3%)
	FVC (%) 75-50	34.0	(79.8%, 45.4%, 21.6%)	37.4	(88.9%, 54.1%, 18.3%)	26.2	(70.7%, 36.9%, 25.4%)
	FVC (%) < 50	12.4	(56.3%, 11.3%, 0%)	17.1	(71.4%, 21.4%, 0%)	10.8	(39.9%, 0%, 0%)
GAP index: DLCO (%)	DLCO (%) > 55	48.3	(89.4%, 65.3%, 32.4%%)	53.4	(95.7%, 77.1%, 29.7%)	42.7	(85.1%, 57.6%, 34.2%)
	DLCO (%) 55-36	29.6	(84.6%, 40.4%, 21.8%)	35.5	(97.4%, 49.1%, 16.4%)	26.2	(71.3%, 31.4%, 31.4%)
	DLCO (%) ≦ 35	14.7	(70.5%, 25.2%, 0%)	16.3	(76.9%, 23.1%, 0%)	12.0	(44.4%, 29.6%, 0%)
	Cannot performed	8.0	(30.0%, 10.0%, 0%)	7.8	(33.3%, 16.7%, 0%)	9.2	(25.0%, 0%, 0%)
GAP plus BMI	Stage I (n=46)	53.4	(100%, 78.8%, 52.0%)	53.3	(100%, 85.7%, 32.3%)	Not reached	(100%, 72.5%, 72.5%)
	Stage II (n=88)	47.2	(91.6%, 60.8%, 34.4%)	48.1	(94.3%, 63.4%, 37.2%)	47.2	(90.1%, 59.4%, 41.0%)
	Stage III (n=72)	24.3	(70.6%, 33.8%, 5.7%)	34.0	(86.5%, 44.8%, 6.9%)	13.9	(53.6%, 22.6%, 6.8%)
	Stage IV (n=23)	12.4	(59.1%, 10.8%, 0%)	14.7	(72.7%, 18.2%, 0%)	11.6	(39.4%, 0%, 0%)

*Median survival (1-yr, 3-yr, 5-yr survival), GAP; Gender-Age-Physiology, FVC; forced vital capacity, DLCO; diffuse capacity of the lung for carbon monoxide, BMI; body mass index, Not reached; more than 50% of the patients were still alive.

-
